# Health, policy and geography: Insights from a multi-level modelling approach^☆^

**DOI:** 10.1016/j.socscimed.2013.05.021

**Published:** 2013-09

**Authors:** Adriana Castelli, Rowena Jacobs, Maria Goddard, Peter C. Smith

**Affiliations:** a Centre for Health Economics, University of York, York YO105DD, UK; b Imperial College Business School and Centre for Health Policy, Imperial College, London, UK

**Keywords:** England, Multilevel models, Small area analysis, Health indicators, Public sector organisations, Policy intervention

## Abstract

Improving the health and wellbeing of citizens ranks highly on the agenda of most governments. Policy action to enhance health and wellbeing can be targeted at a range of geographical levels and in England the focus has tended to shift away from the national level to smaller areas, such as communities and neighbourhoods. Our focus is to identify the potential for targeting policy interventions at the most appropriate geographical levels in order to enhance health and wellbeing. The rationale is that where variations in health and wellbeing indicators are larger, there may be greater potential for policy intervention targeted at that geographical level to have an impact on the outcomes of interest, compared with a strategy of targeting policy at those levels where relative variations are smaller. We use a multi-level regression approach to identify the degree of variation that exists in a set of health indicators at each level, taking account of the geographical hierarchical organisation of public sector organisations. We find that for each indicator, the proportion of total residual variance is greatest at smaller geographical areas. We also explore the variations in health indicators within a hierarchical level, but across the geographical areas for which public sector organisations are responsible. We show that it is feasible to identify a sub-set of organisations for which unexplained variation in health indicators is significantly greater relative to their counterparts. We demonstrate that adopting a geographical perspective to analyse the variation in indicators of health at different levels offers a potentially powerful analytical tool to signal where public sector organisations, faced increasingly with many competing demands, should target their policy efforts. This is relevant not only to the English context but also to other countries where responsibilities for health and wellbeing are being devolved to localities and communities.

## Highlights

• Policies to enhance wellbeing may be targeted at different hierarchical levels such as individual, neighbourhood or region.
• Organisations responsible for implementing such policies also operate in geographically defined administrative boundaries.
• We explore the variation in a range of health-related indicators at a number of geographical hierarchical levels.
• We find evidence of variation between organisations within the same hierarchy and also between hierarchical levels.
• The results direct policymakers in public sector organisations to target their policy efforts where impact may be greatest.

## Introduction

Improving the health and wellbeing of citizens is high on the agenda of most governments and policies aimed at enhancing this key objective can be targeted at a number of different levels such as the individual, neighbourhood, community, locality, local authority, district, region, or national level. For some years, there has been an increasing policy focus in England on the level of community and neighbourhood, culminating most recently in the notion of the “Big Society” which has an emphasis on “localism” and “community” at its core (Lawless, 2011). Typically, health care reform is likely to involve a shift in policy focus to different geographical levels within the health care system and again, the most recent NHS reforms switch attention to smaller geographical areas (NHSCBA, 2012). Many of the public sector organisations (PSOs) responsible for implementing such policies are organised in geographical hierarchies with each organisation tasked with responsibilities that may affect the welfare of individuals within their jurisdiction, either at the hierarchical level where the PSO is positioned, or at lower levels in the hierarchy. Thus there is an interest in knowing where best to target policies in order to improve health and wellbeing. As health and wellbeing is influenced by actions taken not only by PSOs responsible for health care, but also by other bodies who may well operate within different geographical boundaries (Audit Commission, 2009), it is also of interest to explore the scope for organisations to exert an influence on health outside their direct jurisdiction.

At the same time there is a growing body of research that focuses on the influence of area of residence on the health and wellbeing of individuals, over and above the aggregate impact of the characteristics of individuals, although there is considerable debate about both the degree of influence and the nature of the specific mechanisms involved (Macintyre, Ellaway, & Cummins, 2002). Reported associations between area of residence (defined in various ways) and measures of health and wellbeing include cardiovascular disease, coronary heart disease, mental health conditions, and a wide range of health related behaviours (Bell, Wilson, Bissonette, & Shah, 2012; Ellaway, Benzeval, Green, Leyland, & Macintyre, 2012). Disentangling the origin of such variations and the complex relationships between individual and place based characteristics is methodologically and conceptually challenging. Thus, research has focused both on trying to establish the relative role of place (“context”) and that of the individual characteristics of people (“composition”) (Macintyre et al., 2002); as well as moving beyond this dual outlook to recognise the interplay between the two and the “mutually reinforcing and reciprocal relationship between people and place” (Cummins, Curtis, Diez-Roux, & Macintyre, 2007). Whilst acknowledging the complexities of understanding the causal mechanisms at work, the place-based factors, which may have some role in influencing health and wellbeing of individuals include a range of economic conditions, physical conditions, environmental and cultural factors, access to health care resources and indicators of social capital (Kawachi, Subramanian, & Kim, 2008; Macintyre & Ellaway, 2003; Pickett & Pearl, 2001).

In this paper, we bring together the two strands outlined above by exploring the variation in a range of health-related indicators at a number of geographical levels. We do not seek to explain the nature of the mechanisms through which place or area is linked with health, nor do we propose any causal mechanisms through which this might work. Our focus is instead on identifying the potential for targeting policy interventions at appropriate geographical levels. Our rationale suggests where variations in health and wellbeing indicators are greater, there may be more potential for policy intervention targeted at that geographical level to have an impact on the outcomes of interest, compared with a strategy of targeting policy at the levels where relative variations are smaller. Similarly, comparison of the degree of variation between areas, but within the same geographical level, may also serve to focus policy attention where the greatest variation is apparent. In both cases, it is feasible that the patterns of variation may differ according to the specific indicator of health and wellbeing under consideration, which also has implications for the policymaker interested in influencing different aspects of the welfare of citizens. Intervention is therefore justified from three perspectives: first, at the geographical level where variations are larger; second, for PSOs within the same geographic scale where variations are larger; and third, for the specific health indicators where the greatest variation is apparent. Of course, even where little variation exists, intervention may still be appropriate, but our argument is that identification of relative variations can be a guide to targeting policy effort more appropriately.

Whilst we focus in this paper on PSOs and the health and wellbeing of the citizens living in the area for which they are responsible, we do not argue that policies targeted at addressing variations at specific geographical levels are necessarily best undertaken by the PSOs that exist at that level. Actions may be undertaken by PSOs at any level and may be targeted at the entire area for which the PSO is responsible or at specific areas under their jurisdiction. Indeed, as we describe later, it is possible that there are no obvious PSOs at those levels identified as being most appropriate to target. However, since organisations and policy-makers are increasingly facing a range of multiple and competing demands for their attention, we seek to give a signal of where the policy efforts of PSOs at any level in the hierarchy are best targeted.

## Policy background

Major policy shifts in England have given rise to two important issues that can best be understood by applying a geographical lens to the analysis of health and wellbeing. First, there has been an increasing emphasis on the “local” dimension in relation to many aspects of public policy making, including health care; and second, a formal change in the responsibilities of Local Authorities has recently been made in order to reflect their role in influencing the health and wellbeing of local populations.

There has been a local dimension to the structure, organisation and focus of health care services and policy for many years (Exworthy, 1998), reinforced by the Darzi review, which put localities at the heart of driving and delivering change in the NHS (Department of Health, 2009a), and most recently encapsulated by a number of changes which focus on strengthening local power and decision-making. These include the devolution of responsibility and budgets for purchasing health care services to local consortia and to individual GP practices within the consortia and the greater involvement of patients and the public in running these services (Department of Health, 2010a). At the same time, reform of the public health function in England has moved a significant element of the public health function from Primary Care Trusts into local government. Local Authorities have a new duty to promote the health of their population, because “Local government is best placed to influence many of the wider factors that affect health and wellbeing” (Department of Health, 2010b).

In addition to the formal blurring of the boundaries between the jurisdictions and remit of PSOs, the strategy also reflects the shift of geographical focus, “… radically shifting power to local communities” where “Localism will be at the heart of [the] system” (Department of Health, 2010b, p. 4). This builds on developments such as the New Deal for Communities which was “one of the most intensive and innovative area-based initiatives ever introduced in England”, running for a 10 year period from 1998 (Batty et al., 2010, p. 5) and placed communities at the heart of the initiative. The Localism Act encapsulates this strategy, devolving “power, money and knowledge to those best placed to find the best solutions to local needs: elected local representatives, frontline public service professionals, social enterprises, charities, co-ops, community groups, neighbourhoods and individuals” (Department for Communities and Local Government, 2010, p. 2). Structures, organisations and financial arrangements are changing in order to reflect the shared responsibility of health and local government organisations for the wellbeing of their local communities.

The nature of these changes suggest that we should look beyond the usual geographical levels of regional, local authority or health district area level to smaller geographical areas that may be more representative of local communities or neighbourhoods, as well as considering the role of local government agencies, rather than just health agencies, on the health and wellbeing of citizens.

## The geographical hierarchical structure

Our aim is to measure the degree of variation in a group of health indicators at different geographical hierarchical levels. As described earlier, where variations are largest, there may be relatively greater potential for policy intervention targeted at that particular geographical level to have an impact on the outcomes of interest, compared with a strategy of targeting levels at which variations are relatively smaller. This is especially important given the policy trends outlined above.

We take account of the fact that PSOs are often structured such that administrative organisations operate at geographically defined levels, with some organisations being clustered within the boundaries of others, in a hierarchical structure. PSOs are usually tasked with addressing variations in health related outcomes for the populations in the geographical areas for which they are responsible. For example, in England large organisations such as Government Regions and Strategic Health Authorities (SHAs) are at the top, with lower level organisations such as Local Authorities (LAs) and Primary Care Trusts (PCTs) nested within these boundaries and much smaller geographical areas below these.

Two geographical hierarchical structures that are relevant in England's health care delivery are examined in this paper. Before presenting the two structures, we define the various geographical levels that are important for our analysis. Firstly, we consider the lowest level in the two geographical hierarchical structures examined in this paper: these are ‘lower layer super output areas’ (LSOAs) and wards.

LSOA is a geographic hierarchy developed by the Office for National Statistics (ONS) to improve the reporting of small area statistics in England and Wales. These give a spectrum of areas that are consistent in size and whose boundaries do not change over time. LSOAs have been constructed specifically to take into account not only mutual proximity and population size, but also ‘social homogeneity’. Super Output Areas (SOAs) are a cluster of output areas (OAs) used for the 2001 Census. Three layers of SOA were created. We use the lowest possible level, the LSOA. The minimum population of each LSOA is 1000, with a mean population of 1500. There are 32,482 LSOAs in total in England. ONS has increasingly started to report national statistics at this unit of aggregation.

The second small area we consider is the ward, both the electoral ward and standard table ward. Electoral wards are the spatial unit used in England to elect local government councillors in metropolitan and non-metropolitan districts, unitary authorities and the London boroughs. They constitute the lowest administrative units in the UK; further, all other administrative units are built up from electoral wards (Office for National Statistics, 2009a). There are 8797 electoral wards in England. Standard table wards are a further subset of statistical wards, where statistical wards which have less than 1000 residents or 400 households have been merged together for confidentiality issues. 2001 Census standard table wards are those for which the 2001 Census standard tables are available. 7932 standard wards exist in England (Office for National Statistics, 2009b).

The other relevant geographical hierarchies are constituted by local government areas, at which level Local Authorities (LA) operate; Government Office Regions (GOR), which define the political and administrative boundaries within which LAs and other agencies and organisations operate; higher-level health organisations, i.e. Strategic Health Authorities (SHAs), which operate within precise and mutually exclusive GOR boundaries; middle-level health organisations, i.e. Primary Care Trusts (PCTs), which operate within the boundaries of an SHA.

LAs have various administrative and financial responsibilities, which include education, housing, transport, social services, environmental health and in some cases tax collection and, as indicated earlier, they are now taking on major responsibilities for public health. There are 354 LAs in England in our model, across nine Regions (GORs).

SHAs were created in 2002 (National Health Service, 2010a). They are responsible for developing plans for improving health care services in the local area, ensuring that local health care services are of high quality and meet local needs and that priorities set at the national level are implemented locally. There are 28 SHAs defined in our model, although the 2006 structural re-organisations reduced the number to 10. More recently a complete overhaul of the structure of the National Health Service has taken place in England, which has abolished SHAs, but the principle of a higher level authority still remains.

PCTs are geographically defined organisations with the aim of ensuring that local communities' needs are met from the purchasing of health care services through to the direct provision of primary care services to their local populations. They work alongside LAs and other agencies to achieve these objectives. Current policy aims to devolve further the responsibilities of PCTs to smaller commissioning consortia (Department of Health, 2010a). There are 304 PCTs defined in our model.

**Fig. 1:**
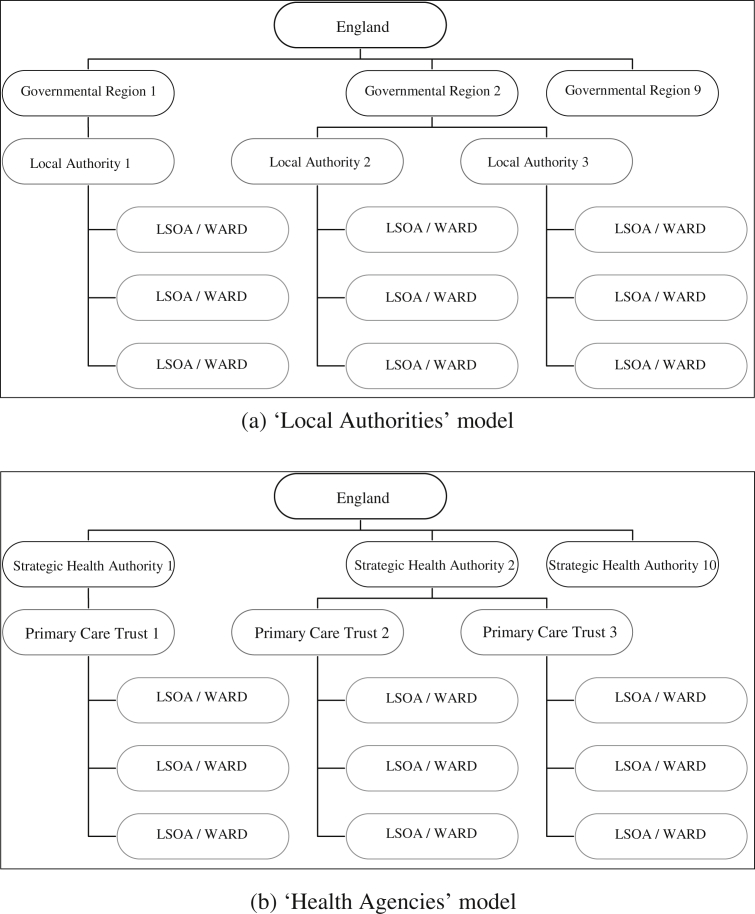
Hierarchical structure and clustering for.

The first geographical hierarchy analysed in this paper - the ‘Local Authorities’ model – has either LSOAs or wards (electoral or standard table, depending on the health indicator) as the smallest unit of analysis; these are uniquely clustered within LAs, which are in turn nested within the GORs, as shown in Fig. 1(a). The second geographical hierarchy – the ‘Health Agencies’ model - has either LSOAs or wards (as above) as the smallest unit of analysis; these are also uniquely clustered within 304 PCTs, which are in turn uniquely clustered within 28 SHAs, as shown in Fig. 1(b).

In both models we choose as our smallest unit of analysis, LSOAs or wards, as we try to capture as far as possible a geographical unit which reflects the policy directions described earlier, i.e. a focus on smaller localities, neighbourhoods or communities. Whilst recognising that area definition is subject to debate (Flowerdew, Manley, & Sabel, 2008; Haynes, Daras, Reading, & Jones, 2007, Haynes, Jones, Reading, Daras, & Emond, 2008) LSOAs and wards are nested within the boundaries of the PSOs of interest to us in this analysis.

The analytical method we use takes account of the geographical hierarchical structure and is described in Methods Section.

## Data

Indicators to capture health, wellbeing and quality of life were developed by the Audit Commission in England and were originally used to help ‘paint a picture’ of the quality of life at LA level (Audit Commission, 2005). The indicators covered ten themes: community cohesion and involvement; economic well-being; transport and access; health and social well-being; community safety; housing; education and life-long learning; environment; culture; and people and place. The focus in this paper is on indicators for health only, although the same methodology can be used to explore all such indicators (Castelli, Jacobs, Goddard, & Smith, 2009). Within the theme ‘Health and social well-being’, we have identified four indicators (mortality, longstanding illness, life expectancy and teenage conceptions). These are described in Table 1, alongside the level at which they are collected and the sign of the indicator which is assumed to be associated with better quality of life (+). (The Audit Commission refers to the set of indicators as “quality of life” indicators. We use this term (or health indicators) because the focus of our paper is not on the usual measures of quality of life of individuals, but on the quality of life and wellbeing experienced by the population of the small areas/communities we consider.)

Our choice of variables is necessarily constrained by data availability at LSOA and ward level, but the indicators selected are used extensively nationally and internationally by governments to assess population health. For instance, mortality and life expectancy are amongst the oldest and most fundamental of health indicators (Department of Health, 2012a, 2012b; OECD 2011; WHO 2012); limiting longstanding illness is collected in the Census for every household in England; and teenage conceptions are associated with poorer outcomes for both young parents and children (Department of Health, 2012a). Moreover, the choice is particularly relevant in the context of our analysis because the indicators are potentially amenable to influence by PSOs, as evidenced by the inclusion of three of the indicators in the Public Health Outcomes Framework for England (Department of Health, 2012b), which states that “attending to these outcomes will require collective efforts…. across public services” (p. 5). This policy approach also highlights that achievement of the outcomes will be “locally-led” and that PSOs in both the NHS and Local Government will have a shared role in helping to achieve the outcomes, and both these aspects are relevant to the focus of our analysis.

Data were drawn from the 2001 Census of Population, Office for National Statistics (and its Geographic Mortality unit) and the Public Health Observatory. Two of the quality of life measures are defined at lower layer super output area (LSOA) and two at ward level, either electoral ward or 2001 Census Standard table ward. Although these data refer to different years, the data collected are considered and analysed in a cross-sectional framework.

Tables 2 and 3 provide descriptive statistics respectively for quality of life indicators defined at LSOA level and those defined at ward level.

Differences in the health care indicators may arise from a wide range of factors and to account partially for this possibility, we control for socio-economic characteristics of the population at these geographical levels. Given our focus on the role of PSOs, we also introduce performance indicators for PSOs, in order to explore whether their differing organisational capabilities are associated with variations in health care indicators at different geographical levels. We describe both groups of factors in more detail below and provide a rationale for our choices.

Socio-economic characteristics of the local population are introduced through the Indices of Multiple Deprivation (IMD) (Office of the Deputy Prime Minister (ODPM), 2004). The IMD is based on the idea that individuals living in a specific area may experience one or more forms of deprivation. Seven domains of deprivation are identified: income deprivation; employment deprivation, health deprivation and disability; education, skills and training deprivation; barriers to housing and services; living environment deprivation and crime. The IMD is recognised as an important indicator of health need, hence it has always been a key component of the formulae used to allocate £85 billion of resources to PCTs annually (Department of Health, 2011). Since the health deprivation and disability domain is either directly or indirectly related to the quality of life measures for health used in our analysis, we exclude the domain specific index for health and disability, in order to avoid potential endogeneity bias. Further, we find a high correlation between the deprivation indices for income and employment, and considering that employment deprivation and longstanding illness are often considered jointly determined (Lindeboom & Kerkhofs, 2009), we do not include the deprivation index for employment.

All indices of deprivation are measured at LSOA level. For health indicators defined at ward level, we construct artificial measures of the domain specific IMD indices. This was done by calculating population (at LSOA) weighted average IMD scores, as follows:

(1) $$x_{tw}=\frac{\sum_{i}p_{i}x_{ti}}{P_{w}}$$

where *x*_*tw*_ is the value of the *t*-th index of multiple deprivation for ward *w*, *x*_*ti*_ is the value of the *t*-th index of multiple deprivation for LSOA *i*, and *p*_*i*_ and *P*_*w*_ are respectively the number of individuals living in LSOA *i* within ward *w* and the total number of individuals living in ward *w*. Descriptive statistics for all deprivation indices are provided in Tables 2 and 3, respectively at LSOA level and ward level.

Finally, we incorporate information on three performance indicators each for Local Authorities and Primary Care Trusts respectively as control variables to pick up organisational effects on our health indicators. The measures of performance we use give a general picture of the PSOs' overall performance via composite scores and also a snapshot of their financial situation and resourcing. PSOs that are generally “performing well”, as measured by the standard indicators that have been used for accountability purposes, are likely to be relatively better placed than those doing less well, to influence the health and wellbeing of their populations. The composite measures also capture aspects of the PSO's performance that may be linked directly to their ability to influence health and wellbeing: for instance, assessments of the quality of the services provided to their population. Whilst there is some debate about the effectiveness of performance assessment as a tool to improve aspects such as health and wellbeing (Blackman et al., 2006), there is a large volume of literature that attempts to investigate the links between performance measurement and health improvement (e.g. Smith, Mossialos, Papanicolas, & Leatherman, 2009) and it is generally well-established that the performance of PSOs is relevant to their ability to perform their functions well. Of course, measured performance as reflected in the indicators will be just one aspect of how well placed PSOs are to tackle problems at local level but in terms of reflecting the organisation's ability, readiness or capacity to make changes that can improve the health and wellbeing of the population, we argue that performance is a useful indicator. We focus in particular on strong financial performance which, although not a sufficient condition for achieving good health outcomes, is likely to be necessary: “Financial stability is both a key objective and a minimum standard for NHS bodies. It provides the essential platform on which to manage and develop patient services in line with the targets/objectives set out in the NHS Plan” (Healthcare Commission, 2004a, p. 1). Finally, we include measures that provide a picture of the overall level of resourcing for PCTs and LAs, on the grounds that those PSOs with more generous funds at their disposal (relative to the level calculated will be required to meet population needs) will find it easier than PSOs with resource constraints, to attend to the health and wellbeing of their population.

Performance indicators (which reflect those in use during the period covered by the data and which may subsequently have been replaced by alternative measures) for LAs are provided within the Comprehensive Performance Assessment (Audit Commission, 2004) and include an overall composite performance score (star rating) which reflects service quality, resource management and governance and an indicator of financial standing, conduct and control. Also included is data on Council Tax (Band D) (Communities and Local Government, 2009) for Local Authorities. This metric offers an indication of the extent to which the LA spends above or below the national standard level of council tax. The grant received by the LA from central government funds is calculated to permit them to deliver a standard mix of services at a standard council tax, thus those Authorities with higher Band D rates are collecting more tax locally and thus potentially may be in a position to spend a greater amount on quantity or quality of services.

Performance indicators for PCTs are provided within their annual assessment (Healthcare Commission, 2004b) and include an overall composite performance score (star rating), largely driven by the degree to which it has achieved key targets such as waiting times, and also a measure of financial performance based on the degree to which it achieved its financial position without the need for unplanned central intervention. We also include PCTs' distance from target (Department of Health, 2009b), which gives the difference between their actual allocation and the resource allocation formula target which is based on an assessment of the amount of resources required to meet population needs (fair funding). The intention is for their actual allocation to converge to target over a number of years. In the interim period, distance to target indicates the extent to which PCTs are over- or under-funded and we would expect that over-funding potentially puts them in a better position to improve the health of their local population by spending more resources.

All the performance indicators are defined at the organisational level, and are to be used as additional control variables in the models. Descriptive statistics are provided in Table 4.

## Methods

We consider a method designed to take into account explicitly the geographical hierarchical structure within which PSOs usually operate, a feature often neglected by conventional regression approaches. We use a multi-level (ML) regression approach to identify at which geographical level the largest variations in our four health indicators exist. The ML analysis allows us to examine simultaneously the effects of different hierarchical area-level variables (Giordano, Ohlsson, & Lindstrom, 2011); to take account of possible correlations of outcomes (non-independence) within middle or higher levels which may otherwise lead to incorrect standard errors and inefficient estimates; to treat middle and higher levels as related; and to examine inter-area variations at each level.

ML models are variations on the familiar regression-based theme in which the error term is decomposed into parts attributable to each level of the hierarchy. The decomposability of the residual variance is particularly important as it allows one to establish at which level of the hierarchy most variation occurs. Further, we are able to identify residual variances at each geographical level, which allows us to isolate geographical areas that are significantly different from the average, after accounting for measures of deprivation and performance indicators.

In our paper, we estimate two multi-level random intercepts models; that is, we allow for variations between areas to be captured by variation in their intercepts only. We do not allow the different areas to have different regression slopes. In particular, we consider two geographical structures as defined in Section The geographical hierarchical structure: the ‘Local Authorities’ model and the ‘Health Agencies’ model. For both models we consider two specifications: one with no explanatory variables – the ‘Basic model’, and one where we control for five domain specific indices of deprivation and performance indicators of PSOs – the ‘Full model’.

We have also considered two further model specifications: ‘Overall need variable’ where we use the overall index of multiple deprivation and ‘Domain specific need variables’ where we control for the five domain specific indices of deprivation only. The results for these two further model specifications are qualitatively similar to the results obtained from the ‘Basic’ and ‘Full’ model and are not discussed. However, results are available on request from the authors.

We present here the two-level random intercept model equations used for the ‘Local Authorities’ model. Firstly, we estimate the ‘Basic model’ where no explanatory variables are added. This is defined as follows:

(2) $$y_{ij}=\beta_{0}+u_{0j}+e_{ij}\;i=1,2,\ldots ,I;\;j=1,2,\ldots ,J$$

where *y*_*ij*_ represents the health indicator in the *i*th small area, within the *j*th Local Authority. The terms *u*_*0j*_ and *e*_*ij*_ represent error components, respectively for the *j*th LA, and for the *i*th small area (LSOA/ward) within the LA. All random errors are assumed to be normally distributed with mean zero and constant variance $\left(\sigma_{u}^{2},\sigma_{e}^{2}\right)$.

Secondly, we add five domain specific indices of deprivation (*x*_*tij*_) and three performance indicators (*z*_*sj*_) for PSOs. Equation (2) becomes:

(3) $$y_{ij}=\beta_{0}+\sum_{t=1}^{5}\beta_{t}x_{tij}+\sum_{s=1}^{3}\beta_{sj}z_{sj}+u_{0j}+e_{ij}\;i=1,2,\ldots ,I;\;j=1,2,\ldots ,J$$

Domain specific indices of deprivation are defined at either LSOA or ward level, whilst the performance indicators are defined at the LA level *j*.

Finally, in the ‘Local Authorities’ model, we also control for differences in governmental regions by including these as 8 dummy variables with the reference dummy being the London region. Regions were included as dummy variables rather than as an additional tier in the ML models because there were so few regions relative to the lower levels.

The equations for the three tier hierarchical structures (the ‘Health Agencies’ model) are presented in Appendix A. No region dummies are introduced in the ‘Health Agencies’ model.

We are particularly interested in eliciting the residual variance that exists at any of the geographical levels, therefore we need to calculate what is known as the Intra-Class (or intra-geographical area) correlation. This measures the degree of similarity of the dependent variable (the particular health indicator under study) amongst members belonging to the same class (i.e. geographical area) (Goldstein, 2003; Snijders & Bosker, 1999). As we use a random intercept model we interpret the intra-class correlation measure as the proportion of total residual variance that is due to differences between groups, in our case geographical areas. In a two level model, with LSOAs at the 1st level and LAs at the 2nd level, an ICC for the LA level (2nd level), in the extreme case, close to 1, would be indicative of a situation where the outcomes of LSOAs are more likely to be influenced by the geographical environment of the LAs they live in. Conversely, an ICC close to 0 would suggest that LSOAs' outcomes are less likely to be influenced by LAs, that is LSOAs “resemble random samples from the whole population” (Merlo, 2003, p. 550). In this latter case, Snijders and Bosker (1999) state that “the grouping is irrelevant for the *Y*-variable [the health indicator in our example] conditional on *X*, and one could have used ordinary linear regression…”(p. 48).

In our model the ICC is used to assess the proportion of total residual variance that can be attributed to the Local Authorities' influence, and is calculated as:

(4) $${\text{ICC}}_{u}=\sigma_{u}^{2}{\left(\sigma_{u}^{2}+\sigma_{e}^{2}\right)}^{-1},\;0<{\text{ICC}}_{u}<1$$

Larger values of ICC_*u*_ are therefore taken to indicate that a large proportion of residual variance in the health indicator may be attributable to the LA level; thus, suggesting a greater potential for intervention to reduce variation at this level (Hauck, Rice, & Smith, 2003; Hauck & Street, 2006).

Although it is assumed that variation at the lowest level is to be considered as being random, the creation of the LSOAs is not. These are in fact generated by merging output areas taking into account measures of population size, mutual proximity and social homogeneity, making it possible that variations across LSOAs are not truly random. Hence, we also calculate the intra-class correlation coefficient for LSOAs. Similar assumptions can be made about the variation at ward level. LSOAs/ward ICCs are calculated as follows:

(5) $${\text{ICC}}_{e}=\sigma_{e}^{2}{\left(\sigma_{u}^{2}+\sigma_{e}^{2}\right)}^{-1},\;0<{\text{ICC}}_{e}<1$$

The computer package Mlwin BETA version 2.02 is used for estimation (Rasbash, Steele, Browne, & Prosser, 2005).

## Results

### Model parameters

The aim of the models is to control for variation at geographical/hierarchical levels. Hence, our primary interest is in the value of the intra-class correlation coefficients for the geographical/hierarchical structures defined above. However, it is worth noting some of the results obtained for the parameters. Tables 5 and 6 present the coefficient estimates for the ‘Local Authorities’ (two-level random intercept) and ‘Health Agencies’ (three-level random intercept) models respectively.

The results for the ‘Local Authorities’ models show that for all health indicators, there are a number of parameter estimates that are significantly positive, implying that areas with higher levels of deprivation are associated with lower levels of health outcome. In particular, a significantly high and positive association is found for the indicator longstanding illness and the deprivation index for income. The result suggests that areas experiencing lower levels of income are associated with higher percentages of households with longstanding illness or disability, as one might expect. Similarly, a high and positive association is found for the indicator teenage conceptions and the deprivation index for income.

Few significant associations are found between the PSO performance indicators and the health indicators. Two health indicators longstanding illness and life expectancy and one of the performance indicators (Band D council tax) for LAs are significant and positive. Our results suggest that areas spending more than they are assessed to require to spend in order to meet their assessed level of need, have a higher proportion of people who suffer from longstanding illness and disability, although with better life expectancy.

Parameter estimates of the regional dummies are significant at the 5% level (not shown here but results are available on request). In the models for mortality and longstanding illness, all government regions tend to show positive coefficient estimates compared to the reference region of London; thus, implying that mortality and longstanding illness are lower in London than anywhere else in England. The opposite result is obtained in the models for life expectancy and teenage conceptions.

In the ‘Health Agencies’ (three-level random intercept) models (Table 6), there are a number of parameter estimates that are significantly positive, and similar to the results obtained in the two-level models. Further, no significant associations are found between the quality of life indicators for health and the performance indicators for PCTs.

Overall, the results produced in both the health and local government hierarchical structures show close similarities and the performance of the relevant PSOs in each structure have little influence on the variations in health indicators. This might suggest that place effects have more influence on such outcomes than the organizational and management capabilities of PSOs.

### Variations in health indicators between geographical levels

**Fig. 2:**
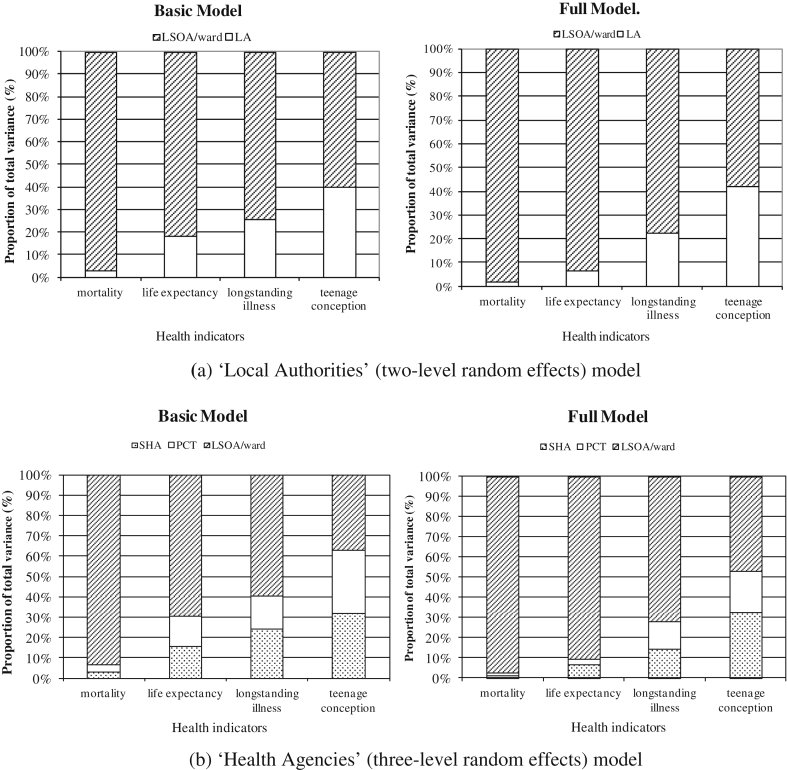
Intra-class correlation coefficients of health indicators.

Intra-class correlations for the ‘Local Authorities’ model (ICCu) are calculated for both the ‘Basic model’ and the ‘Full model’. Estimates of residual variance at LA level for all health indicators are significant at the 5 percent level. A graphical representation of the intra-class correlations attributable to both LAs and LSOAs/wards for the four quality of life indicators is given in Fig. 2(a). Quality of life indicators have been ranked in ascending order of proportion of residual variance at LA level.

In the ‘Basic model’ specification, virtually no variation is detected at the LA level for the standardized mortality measure, with the majority of variation occurring at the LSOA level. Some variation at LA level is detected for the remaining quality of life indicators, especially for teenage conceptions for which the intra-class correlation at LA represents about 40 percent of total residual variance. This suggests that policies targeted at the LA level may have an important role to play in influencing the numbers of teenage conceptions.

We expect that differences in the health indicators may arise from variations in socioeconomic characteristics of the population and we also allow for differences in the performance of Local Authorities to have some influence over their ability to address the needs of their population. To account for this possibility we introduce domain specific measures of deprivation and indicators of LAs' performance in the ‘Full model’. The results obtained do not, however, change significantly from the ‘Basic model’ specification with the least variation in residual variance still attributable to LAs. The exception, as before, is the teenage conception indicator with intra-class correlations slightly increasing after controlling for external factors and performance indicators of the LAs. From both model specifications it appears that the greatest residual variance exists at LSOA/ward level.

Fig. 2(b) provides a graphical representation of the intra-class correlations for all health indicators in the ‘Health Agencies’ model for both specifications analysed. Estimates of residual variance for both SHAs and PCTs for all health indicators are significant at the 5 percent level.

The results in the ‘Basic model’ specification show some variation in health indicators at both SHA and PCT level; about 62 percent of total residual variance in the teenage conception indicator is found at both SHA and PCT combined, this is reduced to about 40 percent for the longstanding illness indicator and to just over 30 percent for the life expectancy measure. We suggest that policies targeted at these levels may be able to exert relatively more influence over these indicators than over standardized mortality, for which the greatest variation occurs at LSOA/ward level.

The intra-class correlation coefficient results obtained in this hierarchical structure change slightly when controlling for socio-economic characteristics of the population and differences in PSO's organisational capabilities. Of particular interest is the change in the intra-class correlations at SHA and PCT level for the life expectancy indicator. Once need is accounted for, just under 90 percent of the residual variance is now found at LSOA/ward level. In general, with the exception of the teenage conception indicator, more than 70 percent of total residual variance is found at LSOA/ward level, a result that suggests that it is at this level that the greatest potential exists for influencing health outcomes. Intervention is therefore justified both at the geographical level where variations are larger and for the specific health indicators where the greatest variation is apparent. Even though PSOs may not readily exist at LSOA/ward level, these results suggest that PSOs at higher levels should target their policies more closely at lower geographic levels and focus relatively more effort on implementing strategies that can reduce the larger variations that are observed at this lower scale. Targeted policies at small area level may not cover the whole PSO jurisdiction.

### Residuals

**Fig. 3:**
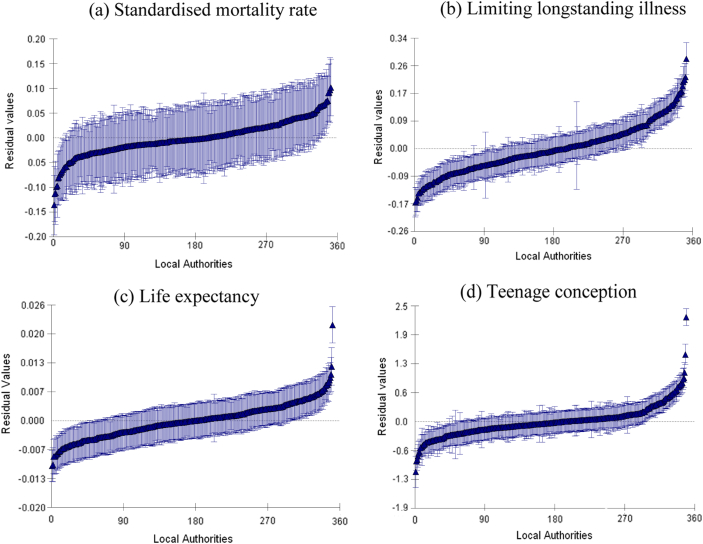
Residual variance for Local Authorities under Full Model.

We analyse the residuals from both the ‘Basic’ and the ‘Full model’ for both the ‘Local Authorities’ and the ‘Health Agencies’ model. We present in Fig. 3 a graphical representation of the residuals from the ‘Local Authorities’ model for the ‘Full model’ specification (other results were qualitatively similar and are not shown). The residual variation from the ‘Full model’ is that which remains after accounting for differences in deprivation and LA performance. We order the residuals for the 354 LAs from lowest to highest values (the dark triangles) and show the 95% confidence intervals surrounding each residual estimate. In order to provide comparable scales across the four health indicators, we produce a normalized measure of dispersion, the coefficient of variation, by dividing all values by each health indicator mean. The ranking allows us to isolate those LAs where the confidence intervals do not overlap or where the residuals represent departures from the overall average predicted by the fixed parameter *β*_0_ and which are therefore significantly different from the average at the 5% level. If we assume that less variation is preferable to more variation given that PSOs are usually tasked with reducing variations, then the LAs facing the biggest challenge on teenage conception, standardized mortality and limiting longstanding illness are clustered on the right (and for life expectancy on the left), while those clustered on the left (on the right for life expectancy) may not be such cause for concern.

We make two related observations about these results. The first relates to the relative position of LAs in terms of the unexplained variation. There are clearly a large proportion of LAs where it is not possible to distinguish their relative ranking given the overlapping confidence intervals. However it is possible to discern a non-negligible group of LAs who exhibit significantly higher levels of variation relative to their counterparts. It is possible to isolate these LAs as candidates for further scrutiny. For these LAs there is clearly far greater scope (compared to other LAs) to focus policies on reducing these higher than expected variations.

The second observation relates to the relative comparison across different health indicators. Confidence intervals for life expectancy overlap much more than for teenage conceptions, since residual values for teenage conceptions span a wider scale. This results in a higher proportion of LAs exhibiting significantly different levels of variation for teenage conceptions and limiting longstanding illness than for standardised mortality and life expectancy and suggests that there is greater scope for LAs to intervene on teenage conception and limiting longstanding illness. We also note one or two LAs that are outliers in terms of the variation in their areas for the teenage conception indicator, which may warrant further investigation by policymakers or the LAs concerned.

Taken together with the results in Fig. 2 from the inter-class correlation coefficients, the residuals in Fig. 3 suggest that teenage conceptions and longstanding illness may benefit more from policies focused at the LA level, and there are significant differences between LAs in the degree of variation attributable at this level. However the majority of the variation is at LSOA/ward level for all health indicators, and therefore policies targeted at this level are likely to have a relatively bigger pay-off.

## Conclusions and policy implications

Our aim was to link together two strands of policy and research: the trend to target policy action to influence health and wellbeing towards smaller, more “local” geographical areas such as communities and neighbourhoods; and the body of research that focuses on the influence of area of residence (“context”) on the health and wellbeing of individuals, over and above the aggregate impact of the characteristics of individuals (“composition”). We also noted that in most public services, administrative organisations are arranged in a hierarchical manner and are defined by geographical administrative boundaries. We have focused particularly on the potential ‘place’ influence on health and the level in the geographical hierarchy at which health policies may best be targeted in order to have an impact.

The identification of the degree of variation in health indicators apparent at each level may be important in terms of focussing policy attention. PSOs are tasked with addressing variations in health and wellbeing and thus for each indicator, where these variations are larger, there may be scope to influence outcomes at that particular level to a greater extent than where the variations are smaller. Although as we have noted, a lack of variation will not always necessarily imply that no intervention is appropriate. We acknowledge that there may be many other factors influencing health outcomes that are outside the control of some PSOs, but we maintain that the existence of such large variations in some areas suggest that PSOs (at different levels) should be aware of them and act so as to reduce or minimise these variations. Although it is not necessarily the case that policies will be most effective if implemented by PSOs that are at the same level at which the greatest degree of variation occurs, the findings give a signal of where the policy efforts of PSOs at any level in the hierarchy are best targeted.

We find that for each indicator, the proportion of total variance attributable to any of the hierarchical levels is relatively robust irrespective of whether the ‘Basic’ or ‘Full model’ is specified. We see that for most indicators the majority of the variation is at the LSOA/ward level. For example in the full specification of the ‘Health Agencies’ model, for the variables mortality, life expectancy, and long-standing illness, 98 percent, 92 percent, and 70 percent of the variation (respectively) is at small area level (LSOA/ward). However, for teenage conceptions it is only 47 percent. Whilst evidence on the existence of small area variation in health indicators is not new (Merlo, Viciana-Fernandez, & Ramiro-Farinas, 2012), our focus is on the possibility that, for instance PCTs and SHAs may be able to exert more influence over teenage conceptions than, say life expectancy, albeit subject to the caveats expressed above.

We find a similar pattern of relative variance across the different health indicators and between hierarchical levels for both Local Authorities and Primary Care Trusts. This finding accords with the increasing recognition that PSOs can influence factors that may be seen as outside their traditional sphere of influence, by virtue of the fact that the actions they take have considerable impact on the lives of their local populations. The results highlight the importance of working across the organisational and administrative boundaries of PSOs. For instance we show that there may be scope for Local Authorities to have some influence over health and well-being in areas that fall outside their statutory responsibilities and we give a flavour of the potential influence that local government organisations could have on measures of health, providing support for partnership working across sector boundaries and the new joint responsibilities for public health. Comprehensive evaluation of the “New Deal for Communities” programme in England has demonstrated that a 10 year area based initiative has been successful in improving the majority of place-related and people-related outcomes in 39 areas of the country, with greater gains made in the place-related outcomes when compared with comparator areas without the New Deal initiative (Batty et al., 2010). The “Total Place” initiative in England has had success with pilot studies aimed at addressing the needs of local communities by “starting from the citizen viewpoint to break down the organisational and service silos” (HM Treasury, 2010, p. 5). This “place-based” approach to local public services has had benefits at all spatial levels and is being rolled-out across the country. Financial mechanisms, which organise all public spending by “place” rather than by individual services and organisations in order to create a “community budget” have been introduced to allow communities to tackle complex issues in their local area. These are to be extended further to neighbourhood level community budgets (Dept for Communities and Local Govt, 2011). Attempts are also being made to assess performance of PSOs on an area basis in terms of their combined impact on quality of life in local areas rather than just within their own boundaries (Audit Commission, 2009). Similarly, the NHS reforms aimed to make the NHS less “insulated and fragmented” by proposing a range of measures to promote cross-boundary working with Local Authorities (Department of Health, 2010a) and passing significant elements of the public health agenda to them (Department of Health, 2010b).

We also offer a further insight by examining the variations in health indicators across PSOs at any one geographical level. We show that it is feasible to identify a sub-set of organisations (LAs in the case of the analysis presented, but the same argument applies to PCTs) for which unexplained variation in health indicators is significantly greater relative to their counterparts. This information can be used to highlight which specific organisations face the biggest challenge in tackling variations in their area and this should guide where action is most needed.

Despite the fact that the largest variations in most indicators are found at the smaller geographical levels in our analysis (LSOA/ward), we are not able to say with any certainty that these signify a “contextual” effect as opposed to a “compositional” effect and indeed, as stated earlier, rather than dichotomous effects, a complex interplay of the relationships is likely to exist. However, given the apparent importance of the small area level emerging from our analysis, what can we say about the role of PSOs and the appropriate focus of policy? Although there may be no obvious PSOs currently located at the small area level, it is vital for those organisations at higher levels with responsibility for improving health and well-being, to be aware of the variation that exists in health indicators at lower levels within their area. The whole thrust of government policy in health and other public sectors in England over the past few years has been to encourage PSOs to become more responsive to local needs and circumstances and to devolve to communities a greater role in decision-making. We are not able to define the mechanisms by which this can be achieved as this is beyond the scope of the paper but note that the English government has created a range of financial and non-financial resources with which to implement local policies and schemes accessible to local community and neighbourhood groups (Department for Communities and Local Government, 2008). Organisations such as “local involvement networks” which aim to harness community level groups in improving health and social care in their area have been developed and supported through the NHS (National Health Service, 2010b).

In conclusion, we have demonstrated that adopting a geographical perspective to analyse the variation in indicators of health at different levels and in particular, focussing on smaller area geographical levels, is feasible if an appropriate methodology, such as multi-level modelling is adopted (Rice & Jones, 1997). Moreover, it offers a potentially powerful analytical tool which is particularly appropriate not only in the UK context, but also in other countries where similar trends can be observed towards devolving responsibilities for health and wellbeing to localities and communities and focussing on collaboration across organisational boundaries (Saltman, Bankauskaite, & Vrangbaek, 2007). Although data availability has restricted the set of health indicators used, we have shown that examining the variation in health indicators that occurs at a number of geographical levels within a model that acknowledges the hierarchical geographical organisation of PSOs, can give some signals about the targeting of policy efforts. We recognise the limitations of our analysis, and do not claim that we can determine the specific policies that should be used by each organisation in order to have maximum effect at each geographical level. However, we think there is merit in drawing together our observations on variations in health and wellbeing indicators with recent policy developments in order to provoke further thought about the appropriate targeting of policy actions. This is especially relevant in the current policy context which has a strong local dimension as well as reflecting shared responsibility for health and wellbeing across jurisdictions and traditional organisational and administrative boundaries. Organisations and policy-makers are increasingly facing a range of multiple, competing demands for their attention and by undertaking a multi-level analysis with a geographical perspective that reflects the hierarchical structure of many PSOs, we provide some useful insights in relation to where their efforts may be best targeted.

## Figures and Tables

**Table 1:** Quality of life indicators (QoL) for health, by level, data source and year.

QoL indicator	Description	Level	Better QoL
Life expectancy at birth	Life expectancy at birth for a ward in 1999–2003 is an estimate of the average number of years a newborn baby is expected to survive if he/she would experience the age-specific mortality rate of that particular ward for that time period throughout his or her life. Data is collected over a number of years, but is used as a cross-section.	Standard ward	+
Teenage conceptions	Number of teenage conceptions at ward level, aggregated for years 2002–2004 due to small numbers, and for confidentiality issues. Data is used as a cross-section.	Electoral ward	−
Standardised mortality ratio	Age-sex standardised mortality ratios, calculated as the ratio of the observed number of deaths in an LSOA to the expected number of deaths, if the LSOA had the same age-sex specific rates as the whole of England. Data are for 2001.	LSOA	−
Households with one or more limiting longstanding illness	Percentage of households in a given LSOA with one or more individuals reporting “limiting long-term illness, health problem or disability that limits their daily activities or work“ (Office for National Statistics, 2004)	LSOA	−

**Table 2:** Descriptive statistics for standardised mortality ratio and households with one or more limiting longstanding illness (*N* = 32,482).

Variable name	Mean	Min	Max	Standard deviation
Mortality	1.122	0.000	7.461	0.474
Longstanding illness	33.449	5.640	70.440	8.367
*Socio-economic factors*				
Income deprivation	0.139	0.002	0.957	0.115
Education, skills and training deprivation	21.691	0.029	99.217	18.777
Barriers to housing and services	21.691	0.276	66.975	10.951
Living environment deprivation	21.691	0.140	93.520	16.728
Crime	0.000	−3.460	3.130	0.839

**Table 3:** Descriptive statistics for teenage conceptions and life expectancy at birth (*N* = a7935; b7932).

Variable name	Mean	Min	Max	Standard deviation
Life expectancy	79.033	65.400	93.400	2.618
Teenage conception	21.136	5.000	168.000	18.283
*Socio-economic factors*				
Income deprivation	0.114	0.010	0.618	0.084
Education, skills and training deprivation	18.557	0.138	92.585	14.639
Barriers to housing and services	23.092	1.053	66.975	11.000
Living environment deprivation	18.037	0.290	76.719	12.680
Crime	−0.286	−3.080	2.210	0.769

a Electoral ward.

b Census 2001 Standard Table ward.

**Table 4:** Public service organisations performance data: descriptive statistics.

Variable name	Description	Obs	Mean	Min	Max	Standard deviation
*Primary Care Trust*						
Star rating	A composite measure of the degree to which PCTs achieved key performance targets, added to an assessment of access and quality of care. Devised by the Dept of Health and Healthcare Commission.	304	1.681	0	3	0.813
Financial management	A measure of the extent to which an organisation has achieved the position shown in its financial plan without the need for unplanned financial support from the Dept of Health. Other intelligence, e.g. audit reports, may also impact on the assessment.	304	0.789	−2	1	0.676
Distance from target	This measures the difference between a PCT's target level of resources calculated to reflect “fair funding “ (which reflects population health needs) and their recurrent baseline allocation of funds.	146	0.034	−5.433	6.622	2.486
*Local Authority*						
Star rating	A composite measure of the degree to which the LA delivered across a range of indicators including service quality, governance and resource use. Devised by the Audit Commission.	353	3.516	1	5	0.980
Financial management	A measure of the quality of the financial standing, conduct and control apparent within an LA, as assessed by auditors during the course of their auditing duties.	353	3.442	2	4	0.601
Band D Council Tax	Amount of council tax payable on a Band D dwelling occupied as a main residence by two adults, before any reductions due to discounts, exemptions or council tax benefit. Each local authority sets a tax rate expressed as the annual levy on a Band D and this decision automatically sets the amounts levied on all types of households and dwellings.	354	1.114	570	1,294	81

**Table 5:** Coefficient estimates for the ‘Local Authorities’ (two-level random intercepts) models.

Mortality	Basic Model		Full Model		Longstanding illness	Basic Model		Full Model	
Constant	1.122*	(0.016)	0.795*	(0.058)	Constant	29.803*	0.682	19.222*	(2.208)
*Socio-economic factors*					*Socio-economic factors*				
Income deprivation	–		1.473*	(0.046)	Income deprivation	–		29.116*	(0.532)
Education, skills and training deprivation	–		−0.002	(0.000)	Education, skills and training deprivation	–		0.125*	(0.003)
Barrier to housing and services	–		0.000	(0.000)	Barrier to housing and services	–		−0.077	(0.003)
Living environment deprivation	–		0.001*	(0.000)	Living environment deprivation	–		−0.051	(0.003)
Crime	–		0.037*	(0.004)	Crime	–		−0.122	(0.055)
*Performance indicators – Local Authority*					*Performance indicators – Local Authority*				
Star rating	–		0.004	(0.005)	Star rating	–		0.007*	(0.002)
Financial management	–		−0.014	(0.008)	Financial management	–		−0.259	(0.281)
Band D council tax	–		0.000	(0.000)	Band D council tax	–		0.008*	(0.002)

Life expectancy	Basic Model		Full Model		Teenage conception	Basic Model		Full Model	
Constant	78.362*	0.206	82.300*	(0.513)	Constant	30.897*	1.868	28.811*	(6.674)
*Socio-economic factors*					*Socio-economic factors*				
Income deprivation	–		−17.148	(0.617)	Income deprivation	–		65.739*	(3.727)
Education, skills and training deprivation	–		−0.002	(0.003)	Education, skills and training deprivation	–		0.329*	(0.018)
Barrier to housing and services	–		0.008*	(0.002)	Barrier to housing and services	–		0.064*	(0.023)
Living environment deprivation	–		−0.017	(0.003)	Living environment deprivation	–		0.014	(0.017)
Crime	–		−0.480	(0.047)	Crime	–		1.962*	(0.367)
*Performance indicators – Local Authority*					*Performance indicators – Local Authority*				
Star rating	–		−0.029	(0.037)	Star rating	–		0.260	(0.504)
Financial management	–		0.037	(0.061)	Financial management	–		−0.483	(0.824)
Band D council tax	–		0.000*	(0.000)	Band D council tax	–		−0.016	(0.006)

* = Statistically significant at the 5% level.

Standard errors in parentheses.

**Table 6:** Coefficient estimates for the ‘Health Agencies’ (three-level random intercepts) models.

Mortality	Basic Model		Full Model		Longstanding illness	Basic Model		Full Model	
Constant	1.118*	(0.016)	0.919*	(0.023)	Constant	33.483*	(0.814)	28.888*	(0.708)
*Socio-economic factors*					*Socio-economic factors*				
Income deprivation			1.587*	(0.070)	Income deprivation	–		27.651*	(0.829)
Education, skills and training deprivation			−0.002	(0.000)	Education, skills and training deprivation	–		0.126*	(0.005)
Barrier to housing and services			0.000	(0.000)	Barrier to housing and services	–		−0.070	(0.005)
Living environment deprivation			0.001*	(0.000)	Living environment deprivation	–		−0.052	(0.004)
Crime			0.031*	(0.007)	Crime	–		−0.127	(0.082)
*Performance indicators – Primary Care Trusts*					*Performance indicators – Primary Care Trusts*				
Star rating			0.010	(0.013)	Star rating	–		0.325	(0.279)
Financial management			−0.011	(0.008)	Financial management	–		−0.291	(0.460)
Distance from target			0.000	(0.002)	Distance from target	–		0.035	(0.075)

Life expectancy	Basic Model		Full Model		Teenage conception	Basic Model		Full Model	
Constant	78.610*	(0.212)	81.163	(0.195)	Constant	23.999*	(2.219)	8.214*	(2.385)
*Socio-economic factors*					*Socio-economic factors*				
Income deprivation			−18.244	(0.917)	Income deprivation			70.893*	(5.776)
Education, skills and training deprivation			0.003	(0.004)	Education, skills and training deprivation			0.320*	(0.028)
Barrier to housing and services			0.006	(0.003)	Barrier to housing and services			0.018	(0.032)
Living environment deprivation			−0.017	(0.004)	Living environment deprivation			−0.023	(0.026)
Crime			−0.382	(0.070)	Crime			2.686*	(0.544)
*Performance indicators – Primary Care Trusts*					*Performance indicators – Primary Care Trusts*				
Star rating			−0.085	(0.098)	Star rating			−0.170	(1.371)
Financial management			0.091	(0.062)	Financial management			0.196	(0.840)
Distance from target			0.004	(0.017)	Distance from target			−0.260	(0.223)

* = Statistically significant at the 5% level.

Standard errors in parentheses.

## Appendix A Regression equations for the three tier hierarchical structure – the ‘Health Agencies’ model

Basic Model

(A1) $$y_{ijk}=\beta_{0}+v_{0k}+u_{0jk}+e_{ijk}\;i=1,2,\ldots ,I;j=1,2,\ldots ,J;k=1,2,\ldots ,K$$

where *y*_*ijk*_ represents the quality of life indicator in the *i*th small area, within the *j*th PCT, within the *k*th SHA. The terms *v*_0*k*_, *u*_0*jk*_ and *e*_*ijk*_ represent error components, respectively for the *k*th SHA, *j*th PCT within the *k*th SHA, and for the *i*th small area within the *j*th PCT within the *k*th SHA. All random errors are assumed to be normally distributed with mean zero and constant variances $\left(\sigma_{v}^{2},\sigma_{u}^{2},\sigma_{e}^{2}\right)$.

Full Model

(A2) $$y_{ijk}=\beta_{0}+\sum_{t=1}^{5}\beta_{t}x_{tijk}+\sum_{s=1}^{3}\beta_{s}z_{sjk}+v_{0ik}+u_{0jk}+e_{ijk}\;i=1,2,\ldots ,I;j=1,2,\ldots ,J;k=1,2,\ldots ,K$$

where *x*_*tijk*_ and *z*_*sjk*_ represent the domain specific indices of deprivation and the performance indicators for PCTs, respectively.

The associated intra-class correlation coefficient used to assess the proportion of total residual variance that can be attributed to the SHA's influence, is calculated in both models as:

(A3) $${\text{ICC}}_{v}=\sigma_{v}^{2}{\left(\sigma_{v}^{2}+\sigma_{u}^{2}+\sigma_{e}^{2}\right)}^{-1},\;0<{\text{ICC}}_{v}<1$$

Larger values of ICC_v_ indicate that a large proportion of residual variance in the quality of life indicator is attributable to the SHA level.

Similarly, for PCTs the ICC_*u*_ attributable to this level is given by:

(A4) $${\text{ICC}}_{u}=\sigma_{u}^{2}{\left(\sigma_{v}^{2}+\sigma_{u}^{2}+\sigma_{e}^{2}\right)}^{-1},\;0<{\text{ICC}}_{u}<1$$

Variation at LSOA level is captured through:

(A5) $${\text{ICC}}_{e}=\sigma_{e}^{2}{\left(\sigma_{v}^{2}+\sigma_{u}^{2}+\sigma_{e}^{2}\right)}^{-1},\;0<{\text{ICC}}_{e}<1$$

## References

[bib1] Audit Commission (2004). Comprehensive performance assessment (CPA) scores. http://archive.audit-commission.gov.uk/auditcommission/subwebs/publications/studies/studyPDF/3282.pdf.

[bib2] Audit Commission (2005). Local quality of life indicators – supporting local communities to become sustainable.

[bib3] Audit Commission, Care Quality Commission, HM Inspectorate of Constabulary, HM Inspectorate of Prisons, & HM Inspectorate of Probation and Ofsted (2009). Comprehensive area assessment; A guide to the new framework Audit commission.

[bib4] Batty E., Beatty C., Foden M., Lawless P., Pearson S., Wilson I. (2010). The new deal for communities experience: A final assessment.

[bib5] Bell S., Wilson K., Bissonette L., Shah T. (2012). Access to primary health care: does neighbourhood of residence matter?.

[bib6] Blackman T., Greene A., Hunter D.J., McKee L., Elliott E., Harrington B. (2006). Performance assessment and wicked problems: the case of health inequalities. Public Policy and Administration.

[bib7] Castelli A., Jacobs R., Goddard M., Smith P.C. (2009). Exploring the impact of public services on quality of life indicators.

[bib8] Communities and Local Government (2009). Local government finance council tax. http://www.local.communities.gov.uk/finance/ct.htm.

[bib9] Cummins S., Curtis S., Diez-Roux A.V., Macintyre S. (2007). Understanding and representing 'place' in health research: a relational approach. Social Science & Medicine.

[bib10] Department for Communities and Local Government (2008). Communities in control: Real people, real power – White paper.

[bib11] Department for Communities and Local Government (2010). Decentralisation and the localism bill: An essential guide. https://www.gov.uk/government/uploads/system/uploads/attachment_data/file/5951/1793908.pdf.

[bib12] Department for Communities and Local Government (2011). Second phase of the local government resource review: Terms of reference. http://webarchive.nationalarchives.gov.uk/20120919132719/http:/www.communities.gov.uk/documents/localgovernment/pdf/1933423.pdf.

[bib13] Department of Health (2009). High quality care for all: Our journey so far. http://www.aemh.org/pdf/NextstageDarzireport.pdf.

[bib14] Department of Health (2009). Health service circular 2002/012-Primary care trusts revenue resource limits 2003/04, 2004/05 & 2005/06. http://webarchive.nationalarchives.gov.uk/+/www.dh.gov.uk/en/Publicationsandstatistics/Lettersandcirculars/Healthservicecirculars/DH_4005021.

[bib15] Department of Health (2010). Equity and excellence: Liberating the NHS. http://www.official-documents.gov.uk/document/cm78/7881/7881.pdf.

[bib16] Department of Health (2010). Healthy lives, healthy people: Our strategy for public health in England. http://https//www.gov.uk/government/uploads/system/uploads/attachment_data/file/151764/dh_127424.pdf.pdf.

[bib17] Department of Health (2011). Resource allocation: Weighted capitation formula. http://https//www.gov.uk/government/uploads/system/uploads/attachment_data/file/152060/dh_124947.pdf.pdf.

[bib18] Department of Health (2012). The indicator guide, health profiles 2012. http://www.apho.org.uk/resource/view.aspx%3fRID%3d116454.

[bib19] Department of Health (2012). Healthy lives, healthy people: Improving outcomes and supporting transparency. http://https//www.gov.uk/government/uploads/system/uploads/attachment_data/file/193619/Improving-outcomes-and-supporting-transparency-part-1A.pdf.pdf.

[bib20] Ellaway A., Benzeval M., Green M., Leyland A., Macintyre S. (2012). “Getting sicker quicker”: does living in a more deprived neighbourhood mean your health deteriorates faster?. Health & Place.

[bib21] Exworthy M. (1998). Localism in the NHS quasi-market. Environment and Planning C: Government and Policy.

[bib22] Flowerdew R., Manley D.J., Sabel C.E. (2008). Neighbourhood effects on health: does it matter where you draw the boundaries?. Social Science & Medicine.

[bib23] Giordano G.N., Ohlsson H., Lindstrom M. (2011). Social capital and health – Purely a question of context?. Health & Place.

[bib24] Goldstein H.B.S. (2003). Multilevel statistical models.

[bib25] Hauck K., Rice N., Smith P. (2003). The influence of health care organisations on health system performance. Journal of Health Services Research and Policy.

[bib26] Hauck K., Street A. (2006). Performance assessment in the context of multiple objectives: a multivariate multilevel analysis. Journal of Health Economics.

[bib27] Haynes R., Daras K., Reading R., Jones A. (2007). Modifiable neighbourhood units, zone design and residents' perceptions. Health & Place.

[bib28] Haynes R., Jones A.P., Reading R., Daras K., Emond A. (2008). Neighbourhood variations in child accidents and related child and maternal characteristics: does area definition make a difference?. Health & Place.

[bib29] Healthcare Commission (2004). Indicator listing for PCTs. http://ratings2004.healthcarecommission.org.uk/Trust/Indicator/indicatorDescriptionShort.asp%3findicatorId%3d4001.

[bib30] Healthcare Commission (2004). Performance ratings. http://ratings2004.healthcarecommission.org.uk/.

[bib31] HM Treasury, Communities and Local Government (2010). Total place: A whole area approach to public services. http://www.hm-treasury.gov.uk/d/total_place_report.pdf.

[bib32] Kawachi I., Subramanian S.V., Kim D. (2008). Social capital and health.

[bib33] Lawless P. (2011). Big society and community: lessons from the 1998–2011 new deal for communities programme in England. People, Place and Policy Online.

[bib52] Lindeboom M., Kerkhofs M. (2009). Health and work of the elderly: subjective health measures, reporting errors and endogeneity in the relationship between health and work. Journal of Applied Econometrics.

[bib34] Macintyre S., Ellaway A., Kawachi I., Berkman L. (2003). Neighbourhoods and health: an overview. Neighbourhoods and health.

[bib35] Macintyre S., Ellaway A., Cummins S. (2002). Place effects on health: how can we conceptualise, operationalise and measure them?. Social Science & Medicine.

[bib36] Merlo J. (2003). Multilevel analytical approaches in social epidemiology: measures of health variation compared with traditional measures of association. Journal of Epidemiology & Community Health.

[bib37] Merlo J., Viciana-Fernandez F.J., Ramiro-Farinas D. (2012). Bringing the individual back to small-area variation studies: a multilevel analysis of all-cause mortality in Andalusia, Spain. Social Science & Medicine.

[bib38] National Health Service (2010). Authorities and trusts. http://www.nhs.uk/NHSEngland/thenhs/about/Pages/authoritiesandtrusts.aspx.

[bib39] National Health Service (2010). Links. http://nhs.uk/NHSEngland/links/Pages/links-make-it-happen.aspx.

[bib40] NHS Commissioning Board Authority (2012). Emerging clinical commissiong group configuration: Names, geography and constituent members. http://www.england.nhs.uk/wp-content/uploads/2012/05/board-item5-310512.pdf.

[bib53] Office of the Deputy Prime Minister (ODPM), Index of Multiple Deprivation (2004). Measure of multiple deprivation at small area level made up of seven domains.

[bib41] Office for National Statistics (2004). Neighbourhood statistics. http://neighbourhood.statistics.gov.uk/.

[bib42] Office for National Statistics (2009). Electoral wards/divisions. http://www.statistics.gov.uk/geography/electoral_wards.asp.

[bib43] Office for National Statistics (2009). Statistical census table ward. http://www.ons.gov.uk/ons/search/index.html%3fnewquery%3dStatistical+Census+Table+Ward.

[bib44] Organisation for Economic Co-operation and Development (OECD) (2011). Health at a glance 2011: OECD indicators.

[bib45] Pickett K.E., Pearl M. (2001). Multilevel analyses of neighbourhood socioeconomic context and health outcomes: a critical review. Journal of Epidemiology & Community Health.

[bib46] Rasbash J., Steele F., Browne W.J., Prosser B. (2005). A user's guide to Mlwin. http://www.bristol.ac.uk/cmm/software/mlwin/download/manuals.html.

[bib47] Rice N., Jones A. (1997). Multilevel models and health economics. Health Economics.

[bib48] Saltman R., Bankauskaite V., Vrangbaek K. (2007). Decentralization in health care: Strategies and outcomes. http://www.euro.who.int/__data/assets/pdf_file/0004/98275/E89891.pdf.

[bib49] Smith P., Mossialos E., Papanicolas I., Leatherman S. (2009). Performance measurement for health system improvement: experiences, challenges and prospects.

[bib50] Snijders T.A.B., Bosker R.J. (1999). Multilevel analysis: An introduction to basic and advanced multilevel modeling.

[bib51] World Health Organization (2012). World health statistics 2012. http://www.who.int/gho/publications/world_health_statistics/EN_WHS2012_Full.pdf.

